# Postoperative pain intensity and incidence following single visit root canal treatment with different obturation techniques: a randomized clinical trial

**DOI:** 10.7717/peerj.13756

**Published:** 2022-07-27

**Authors:** Aliye Koçer, Hicran Dönmez Özkan, Tugba Turk

**Affiliations:** 1 Department of Endodontics, Aydın Adnan Menderes University, Efeler, Aydın, Türkiye; 2 Department of Endodontics, Ege University, Bornova, İzmir, Türkiye

**Keywords:** Root canal obturation, Endodontics, Postoperative pain, GuttaCore, GuttaFlow2, Cold lateral compaction, Analgesic intake, Obturation technique

## Abstract

**Background:**

There are few studies in the literature about the effect of obturation techniques on postoperative pain. Besides, GuttaFlow2 was used for the first time in this study regarding postoperative pain. This study aimed to compare the postoperative pain levels and incidence following single-visit root canal treatment with different canal filling techniques; cold lateral compaction (CLC), thermoplasticised solid-core carrier (GuttaCore) based filling and cold free-flow compaction (GuttaFlow2) technique.

**Methods:**

The patients (*n* = 93) having single-rooted teeth with a single canal diagnosed with asymptomatic irreversible pulpitis or single-rooted vital teeth with a single canal requiring endodontic treatment because of prosthetic reasons were enrolled in this study. Patients were randomized into three groups (*n* = 31) according to the obturation technique. A single operator performed all the treatments in a single visit. Data on obturation levels, postoperative pain and analgesic intake frequency were recorded at postoperative 6, 12 and 24 h and daily afterward until the 7^th^ day. Postoperative pain was measured by visual analogue scale (VAS). The date were statistically analyzed with chi-squared tests (for the analyses of the categorical data), the nonparametric Kruskal-Wallis test (for the comparisons of VAS score) and with the Friedman test (for the assessments of the changes in VAS scores over time).

**Results:**

The GuttaCore group recorded the higher pain levels, except first 12 h, on the other hand, the GuttaFlow2 group recorded the lower pain levels at all time periods. Significant differences occurred among the groups during the first 4 days (*p* < 0.05), except at 12 h (*p* = 0.054). The patients in the CLC and GuttaFlow2 groups did not need to use the prescribed analgesic; however, one patient in the GuttaCore group used it once.

**Conclusions:**

Postoperative pain levels following root canal therapy were affected by the obturation technique especially first 4 days following obturation.

## Introduction

Following root canal treatment, 3% to 58% of patients report postoperative endodontic pain (Alonso-Ezpeleta et al., 2012; Sathorn, Parashos & Messer, 2008), which may start within a few hours after the procedure and can pose several problems for both patients and clinicians (Alonso-Ezpeleta et al., 2012; Wang et al., 2010). Postoperative endodontic pain may be due to acute inflammation caused by chemical, mechanical or microbial injuries in periapical tissues (Wang et al., 2010). Root canal obturation is suspected to be one of the determinants of postoperative pain (Sathorn, Parashos & Messer, 2008; Wang et al., 2010; Kandemir Demirci & Çalişkan, 2016).

Root canal filling materials are in direct contact with vital tissues, making them true implant materials with specific biological, mechanical and chemical features. Cold lateral compaction (CLC) is the most common method of root canal obturation because of its simplicity, reliability, low cost, ease of dimensional control and retreatment procedures, and suitability for post-space preparation (Orstavik, 2010; Johnson et al., 2016). However, it is time-consuming, lacks penetrability in root canal irregularities, causes voids in the obturation mass and increases the risk of vertical root fracture (Johnson et al., 2016).

In warm vertical compaction techniques, the obturation material mass becomes more homogeneous and adapts better to root canal irregularities compared to CLC (Lea et al., 2005; Gutmann et al., 1993). Thermafil (Tulsa Dental Products, Tulsa, OK, USA), as one of the warm obturation techniques, has superior features to CLC; however, its major limitation arises from the use of metal or plastic carriers coated with alpha-phase gutta-percha (Gutmann et al., 1993). In order to overcome these challenges, the manufacturer released a third-generation carrier (GuttaCore obturator; Dentsply, Charlotte, NC, USA; Tulsa Dental Specialties, Tulsa, OK, USA), which consists of cross-linked gutta-percha instead of plastic as the core material (Nevares et al., 2015). In 2004, GuttaFlow (Coltène/Whaledent, Cuyahoga Falls, OH, USA), which is a poly-dimethylsiloxane based, cold, flowable, self-curing, shrinkage-free root canal filling material combines gutta-percha particulates (smaller than 30 μm), nanosilver particles and sealer was introduced (Zielinski, Baumgartner & Marshall, 2008). It is in a single dose capsule that is injected after mixing. Later on, in 2012, GuttaFlow2 (Coltene/Whaledent, Cuyahoga Falls, OH, USA) was launched in the market as an improved version of GuttaFlow (Collado-González et al., 2017). This new version consists of similar components but in modified proportions. Besides, Guttaflow2 is provided in a premixed form to improve consistency of mixing and facilitate its use. Furthermore, manufacturer claims better sealing and good adaptability, due to its increased flowability and slight expansion (0.2%) on setting. It is applied by a no-compaction obturation technique called the flowable gutta-percha obturation technique or cold free-flow compaction technique (Mohan Kumar et al., 2012; Punjabi, Dewan & Kochhar, 2017). GuttaFlow2 is hypothetically considered to cause less pain after obturation than AH Plus (Dentsply DeTrey; GmbH, Konstanz, Germany) due to its better biocompatibility (Saygili et al., 2017). To the best of our knowledge, post-treatment endodontic pain following the use of GuttaFlow2 on have not yet been studied.

In this context, the present study aimed to evaluate the effects of three different root canal obturation techniques on postoperative pain following single-visit root canal treatment in patients with asymptomatic irreversible pulpitis or vital pulps. The null hypothesis of this study was that the type of obturation technique would not change the incidence and intensity of post-treatment endodontic pain.

## Materials and Methods

The minimum estimated sample size was calculated using the G*Power 3.1 software (Heinrich Heine, University of Düsseldorf, Düsseldorf, Germany) based on previous research data (Alonso-Ezpeleta et al., 2012). An alpha-type error of 0.05 and a beta power of 0.90 was stipulated, and the sample volume was determined to be at least 84 (28 per group). A total of 93 patients (31 teeth per group) were enrolled in this study, considering the potential patient dropout rate and, also, to increase the statistical power of this study.

All procedures performed in studies involving human participants were in accordance with the ethical standards of the institutional and/or national research committee and with the 1964 Helsinki declaration and its later amendments or comparable ethical standards. The research protocol was registered in the ClinicalTrials.gov database (NCT/04228913), and the study was approved by the Clinical Research Ethics Committee of Aydın Adnan Menderes University, Aydın, Turkey (Protocol ADUDHF2017/21). Informed consent was obtained from all participants included in the study.

The patients were selected from those referred to the Hospital of Aydın Adnan Menderes University of Dental Faculty, for root canal treatment from January 2018 to January 2019. All patients meeting the inclusion criteria were invited to participate in the study. Ninety-three systematically healthy patients (59 females and 34 males) aged between 18 to 65 years met the criteria and agreed to participate in the study. Patients presenting with single-rooted teeth with a single root canal diagnosed with asymptomatic irreversible pulpitis (teeth containing vital pulps, that were exposed due to trauma or caries) or single-rooted vital teeth with a single root canal requiring root canal treatment due to prosthetic reasons were included. During the removal of caries in asymptomatic irreversible pulpitis teeth large pulpal exposure may occur. In some cases, adequate haemostasis may not be achieved in 2–3 min. (Aslan & Dönmez Özkan, 2021). In this study, the other identifiers of this diagnosis were profuse bleeding of the pulp after exposing and an inability to achieve haemostasis. The other inclusion and exclusion criteria are listed in Table 1. All participants were informed accordingly and signed a written informed consent form.

**Table 1:** Inclusion and exclusion criteria. Inclusion/exclusion criteria of the participants.

Inclusion criteria	Exclusion criteria
Patients between 18–65 years of ages Patients who agree to participate this study	Patients who refuse to participate this study
Patients diagnosed with asymptomatic irreversible pulpitis caused by deep carious lesion with the single rooted teeth with single canal and patients had been referred for endodontic therapy for prosthetic reasons with the single rooted teeth with one canal Teeth are asymptomatic	Medically compromised patients (with immunosuppressive/systemic diseases, patients on medications) Negative response to thermal and electrical pulp tests
Patients had not used any analgesic, anti-inflammatory and antibiotic drugs over the last 7 days Patients who had good oral hygiene	The presence of advanced periodontal disease (probing depth >4 mm) The presence of pre-operative percussion and palpation in the relevant tooth The presence of open apex, presence of calcification, presence of resorption
Prolonged positive response to cold test (EndoIce; Coltene/Whaledent Inc, Cuyahoga Falls, OH, USA) and electric pulp tester (Parkell, Brentwood, NY, USA)	Patients who had multiple teeth requiring endodontic treatment
Patients who had healthy periapical tissues (confirmed with periapical radiography)	Patients with allergic sensitivity to materials and agents that should be used during the root canal treatment Patients who had allergic sensitivity to local anesthetics Patients who had systemic or allergic sensitivity for the NSAIDs
	Pregnant patients and patients in lactation period

### Treatment protocol

To prevent bias, all treatments were performed by the same clinician (A.K.) who has 5 years of clinical experience. Each root canal was treated in a single visit to minimize the number of procedures and potential pain-related effects of the intracanal medicaments. During the diagnostic examination, periapical radiographs were obtained by the long-cone paralleling technique using a phosphor plate system (Vistascan Mini Easy; Dürr Dental, Bietigheim-Bissingen, Germany) under standard exposure conditions. Thermal (Endo-Ice; Parkell, Inc., Edgewood, NY, USA) and electric pulp tests (Parnell, Inc., Maplesville, AL, USA) were performed to verify the sensibility of the related tooth.

Anaesthesia was achieved with 4% articaine hydrochloride containing 1:100,000 adrenalin. None of the patients required additional anaesthesia. Then, the rubber dam (Coltène/Whaledent Inc, Cuyahoga Falls, OH, USA) was applied for isolation. Following the access cavity preparation, pulpal bleeding was observed to confirm the vitality of the related tooth. Subsequently, working length was determined to be 1 mm shorter than the value (zero) indicated by the electronic apex locator (Raypex 6; VDW GmbH, Munich, Germany) and confirmed radiographically. Apical patency was established with a size 10 K-file, and initial shaping was performed using up to a size 20 K-file. Then, the root canals were prepared using a nickel–titanium rotary instrument (ProTaper Next; Dentsply Sirona, Ballaigues, Switzerland) with up to an X3 file (Dentsply Sirona, Ballaigues, Switzerland) in a crown-down manner at the speed and torque settings recommended by the manufacturer. Additional instrumentation was performed, if necessary, using X4 and X5 files (Dentsply Sirona, Ballaigues, Switzerland). After each file, root canals were irrigated with 2 mL of 5% sodium hypochlorite (NaOCl) (Sigma-Aldrich, St. Louis, MO, USA); a total of 10 mL of 5% NaOCl was used for the irrigation of each canal. In all irrigation procedures, a 27-gauge notched-tip irrigation needle (Endo Eze; Ultradent Products, Inc., South Jordan, UT, USA) was used, which was set to be 2 mm shorter than the working length. In the final irrigation, 3 mL of 17% EDTA (Sigma-Aldrich), 3 mL of 5% NaOCl and 3 mL of distilled water were used, respectively. Then, the root canals were dried with sterile paper points (DiaDent, Seoul, Korea), and the participants were randomly assigned one of the three study groups by using online software (http://www.random.org). To prevent bias, a dental assistant who was blinded to the study procedures performed the allocation. Root canal fillings were performed as follows:

**Group 1** (CLC technique): Control radiography was performed at the working length with a master gutta-percha cone (Dentsply Sirona, Ballaigues, Switzerland). AH Plus was prepared according to manufacturer’s recommendations Next, the master gutta-percha cone was immersed in AH Plus root canal sealer (Dentsply Sirona, Ballaigues, Switzerland) and placed in the root canal within the designated working length. Accessory gutta-perchas (DiaDent, Seoul, Korea) were used until the 25# spreader penetrated no more than 1–2 mm from the root canal orifice. Excess gutta-percha cones were removed using a warm excavator.

**Group 2** (Cold free-flow compaction technique): Control radiography was performed with a master gutta-percha cone (Dentsply Maillefer, Ballaigues, Switzerland) fitted to the working length. Then, GuttaFlow2 was dispensed on the mixing paper as per the manufacturer’s recommendations. The master cone was immersed in this mixture and placed into the root canal to coat GuttaFlow2 to the root canal walls. Next, the master cone was recoated and applied with reciprocating movements to the root canal at the working length. Excess gutta-percha cone was removed using a warm excavator.

**Group 3** (Thermoplasticised solid-core carrier technique): A size verifier matching the last file was used to confirm the adaptability. Periapical radiography was performed to ensure its adaptation to the working length. Light coating of AH Plus was applied with absorbent sterile paper points which was inserted to the coronal half of the root canal system, the second sterile paper point, was placed to the root canal to remove any excess of sealer. After the root canal walls were lightly coated with the sealer, a GuttaCore Obturator were heated in a GuttaCore Obturator Oven (Dentsply Tulsa Dental, Tulsa, OK, USA) as recommended by the manufacturer. The heated GuttaCore obturator was placed in the canal at the previously determined working length by a single and slow movement without rotation and excessive pressure and held firmly with one finger. Then, the shaft and handle of the GuttaCore was severed at the cementoenamel junction using a diamond bur in a high-speed handpiece under copious water spray.

Directly after the root canal obturation, the coronal access cavities were restored with direct adhesive build-up using a composite resin material (Single Bond Universal; 3M ESPE, St. Paul, MN, USA) in all groups.

### Postoperative pain evaluation

After the treatment, two forms (one form for VAS score and the other for analgesic intake) were given to the patients. Patients were instructed on how to determine and mark the postoperative pain level on the VAS scale which was marked in every mm from 0 to 100, at postoperative 6, 12 and 24 h, and then daily until the 7th postoperative day. The VAS scale varies from 0 to 100 mm, where 0 mm corresponds to ‘no pain’, and 100 mm indicates ‘unbearable pain’ (Jabeen & Khurshiduzzaman, 2014; Nekoofar et al., 2015; Ng et al., 2004). Besides marking the VAS line at a point corresponding to their pain level, the patients were also asked to write the value on the table below the scale. Ibuprofen (Brufen; Abbott Laboratories, Abbott Park, IL, USA) was prescribed (400 mg) as an analgesic and anti-inflammatory drug. Patients were advised to take it orally when they encountered severe pain, as previously suggested by Keskin et al. (2017). Patients who took this medication were asked to record the day and time of the intake. Patients were phoned for seven consecutive days at previously scheduled times to obtain information regarding their postoperative pain levels and analgesic need.

Two independent observers (experienced endodontists) examined the periapical radiographs to evaluate the root canal obturation levels. The obturation length at a distance of within 0–2 mm of the radiographic apex was considered ‘acceptable’, a distance of more than 2 mm from the radiographic apex was considered ‘short’ and an extension beyond the radiographic apex was considered ‘overfilled’ (Sjögren et al., 1990).

### Statistical analysis

Statistical analysis was performed using SPSS 24.0 (IBM Corp., Armonk, NY, USA) at *p* < 0.05. Descriptive statistics were given as percentages, numbers and means with standard deviations. The collected data were not normally distributed according to the Lilliefors-corrected Kolmogorov–Smirnov test. Chi-square tests were used for the analyses of the categorical data. Mann–Whitney U tests were performed to compare two independent samples, and the Wilcoxon test was used to compare two dependent samples. The Kruskal–Wallis H test was performed to compare three independent samples (VAS scores). In the *post hoc* analysis, the pairwise comparison module of SPSS was used. The Friedman test was conducted to evaluate the pain score during follow-up. Consistency between observer decisions was assessed by Cohen’s kappa test. The level of statistical significance was set to 5%.

## Results

**Figure 1:**
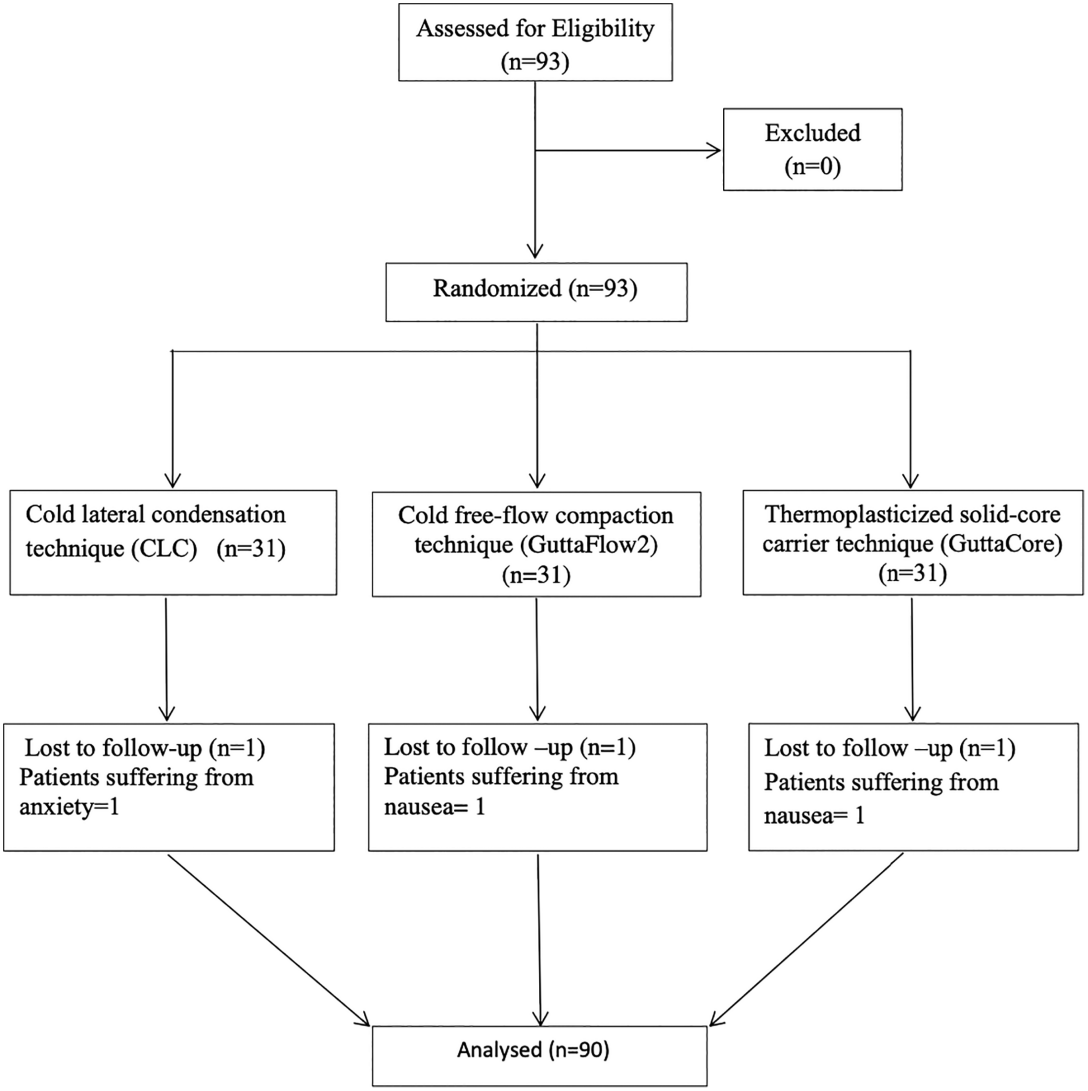
Flowchart. A flowchart of the participants.

One patient from each group was excluded from the study, as their endodontic treatment could not be completed in a single visit as a consequence of strong nausea or anxiety. In total, 90 root canal treatments were performed on 90 patients (Fig. 1). The demographic variables and clinical features of the patients are summarized in Table 2. All patients were reachable by phone; there were no patient dropouts. None of the patients experienced flare-up or swelling following the root canal treatment. There was no significant difference in age (*p* = 0.098) and gender distribution (*p* = 0.824) between the groups.

**Table 2:** Features of the patients. Demographic and clinical features of the patients.

	Sex, *n* (%)		Mean age	Patient (*n*)	Maxillary incisors and canines	Maxillary premolar	Mandibular incisors and canines	Mandibular premolar
	Female	Male						
CLC	20 (66.7)	10 (33.3)	37.90	30	12	5	2	11
GuttaFlow2	18 (60.0)	12 (40.0)	43.07	30	12	3	7	8
GuttaCore	20 (66.7)	10 (33.3)	37.53	30	7	2	3	18
Total	58	32		90	31	10	10	39
								

**Figure 2:**
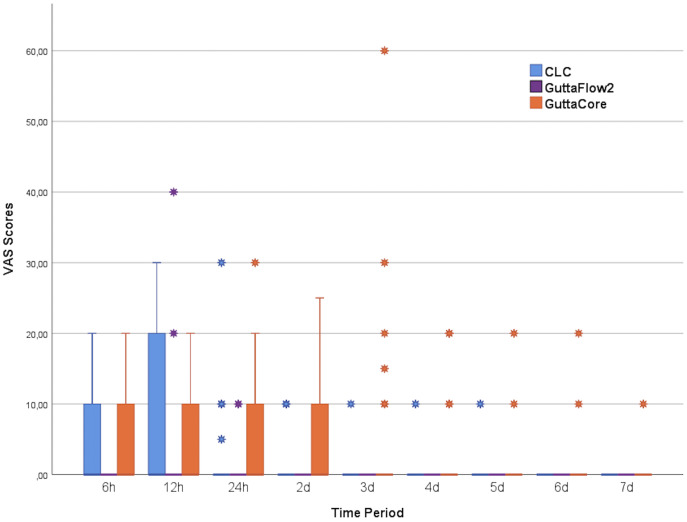
Vas scores of each group. VAS scores of each group at all study time points are shown on the box plot chart.

**Figure 3:**
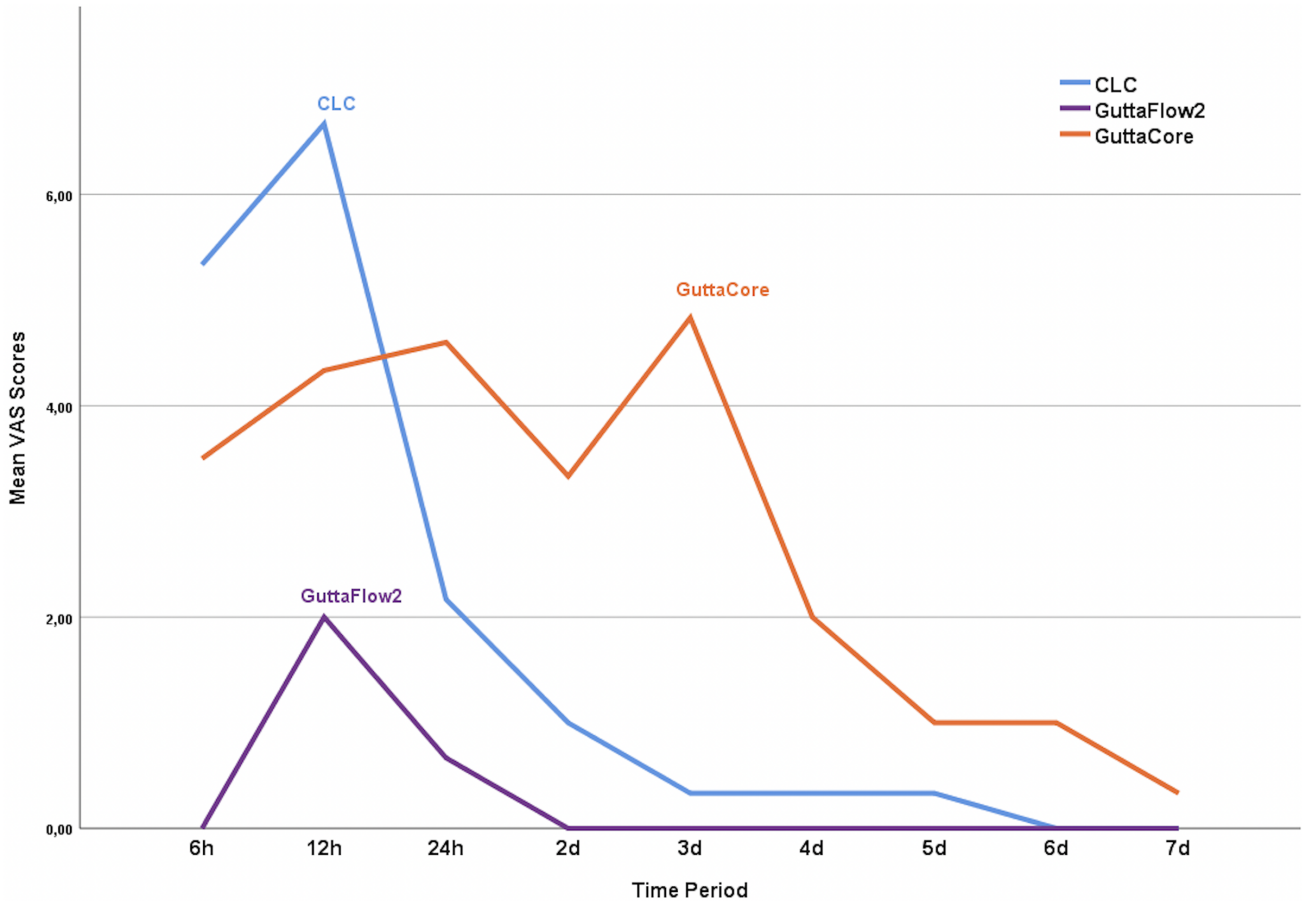
The changes in the Vas score over time. The changes in the Vas score over time in each group are shown on the mean plot chart.

The minimum and maximum postoperative pain scores of the patients during follow-up are displayed in Fig. 2. VAS scores were decreased over time for all groups (Fig. 3). Significant differences in the VAS scores were observed among the groups postoperative 6 h (*p* = 0.023) and 24 h (*p* = 0.004), as well as on the 2nd (*p* = 0.002), 3rd (*p* = 0.003) and 4th (*p* = 0.023) days, on the other hand, there were no significant differences at other time points (*p* > 0.05).

VAS scores in the GuttaFlow2 group at postoperative 6 h were lower than those in the CLC group (*p* = 0.023). No significant difference in VAS scores was detected between the GuttaCore group and the other groups at postoperative 6 h (*p* > 0.05). At postoperative 24 h (*p* = 0.004), day 2 (*p* = 0.002) and day 4 (*p* = 0.029), the VAS scores in the GuttaFlow2 group were lower than those in the GuttaCore group and there was no significant difference in the VAS scores between the CLC group and the other groups (*p* > 0.05). On the 3^rd^ day, VAS scores were higher in the GuttaCore group than in the GuttaFlow2 (*p* = 0.004) and CLC (*p* = 0.018) groups, but there was no significant difference between the CLC and GuttaFlow2 groups (*p* = 0.08).

According to the Friedman test, no differences existed in the VAS scores of GuttaFlow2 group over time (*p* = 0.082, df:8) (*p* = 0.082, df:8). However, in the CLC (*p* = 0.00, df:8) and GuttaCore groups (*p* = 0.00, df:8), differences existed in the VAS scores of each tested method over time. In the CLC group, there were significant differences at the postoperative 6^th^ h (*p* = 0.194) and on days 2 (*p* = 0.009), 3 (*p* = 0.004), 4 (*p* = 0.004), 5 (*p* = 0.004), 6 (*p* = 0.004) and 7 (*p* = 0.004). In the GuttaCore group, there was significantly higher VAS scores at the postoperative 12th, 2nd, 3rd days compared to the postoperative 5th, 6th, 7th days (*p* < 0.05). No significant difference was found at the 4th postoperative day and between days 1 (*p* = 0.051), 2 (*p* = 0.241), 3 (*p* = 0.168), 5 (*p* = 0.276), 6 (*p* = 0.276) and 7 (*p* = 0.102).

At all time-points, GuttaFlow2 group recorded lower pain levels compared to the other groups. Besides, in the GuttaFlow2 group, 86.7% of the patients indicated no pain throughout the study. The remaining 13.3% experienced postoperative pain, which was highest at postoperative 6 h (10.0%) and 12 h (13.3%). None of these patients experienced pain after the 2^nd^ day. Sixty percent of the patients in the CLC group declared no pain throughout the study. The remaining 40.0% had pain, which was most severe at 6 (36.7%) and 12 h (40.0%) after obturation. These analyses showed that both the number of patients with pain and the pain severity decreased from postoperative 12 h. None of the patients declared pain after the 6^th^ postoperative day. In the GuttaCore group, 56.7% of the patients reported no pain throughout the study. For the other patients in this group, the pain rates were calculated as 40.0%, 43.3% and 40.0% for postoperative 6 h, 12 h and day 1, respectively. In this group, both the number of patients with pain and the pain severity began to decline after the first postoperative day. Only one patient declared mild pain on the 7^th^ postoperative day.

The patients in groups CLC and GuttaFlow2 did not need to use the prescribed analgesic. One patient in the GuttaCore group used it once, but there was no difference in terms of analgesic intake among the groups (*p* > 0.05).

There was no relationship between gender and the postoperative pain among the tested groups (*p* > 0.05). However, there was an inverse correlation between age and postoperative pain severity (*p* < 0.05). Analyses of the relationship between age and pain levels revealed a significant correlation at postoperative 6 h (*p* = 0.001, *r* = −0.344), 12 h (*p* = 0.037, *r* = −0.220), day 2 (*p* = 0.002, *r* = −0.324) and day 3 (*p* = 0.007, *r* = −0.282); less pain was observed with increasing age. At the other time points, there was an inverse but insignificant correlation between postoperative pain severity and increasing age.

There was a good correlation (Cohen’s kappa coefficient 0.738–0.854) among the intra-observers and inter-observers in assessing the root canal obturation lengths. In the CLC group, 26 (86.7%) teeth were considered acceptable, and four (13.3%) were overfilled. In group GuttaFlow2 group, the number of teeth classified as acceptable was 21 (70.0%), and nine (30%) were overfilled. The GuttaCore group had eight (26.7%) teeth regarded as acceptable, and 22 (73.3%) were overfilled (Table 3). *Post hoc* analysis revealed that among the three groups, the overfilling (extrusion) rate was significantly higher in the GuttaCore group, whereas the acceptable filling rate was significantly higher in the CLC group. Material overfilling led to increased pain severity on the 1^st^ (*p* = 0.003) and 2^nd^ (*p* = 0.005) postoperative days (Table 4).

**Table 3:** Obturation levels. Root canal obturation levels for the tested obturation techniques.

	Short (>2 mm from apex)		Acceptable (0–2 mm from apex)		Overfilled		χ^2^	*p*
	N	%	N	%	N	%		
CLC	–	–	26	88.7	4	13.3	24.218	<0.001
GuttaFlow2	–	–	21	70	9	30		
GuttaCore	–	–	8	26.7	22	73.3		

**Table 4:** Postoperative pain distribution. Postoperative pain distribution as per obturation lengths at different time points.

	Acceptable (*n* = 55)	Overfilled (*n* = 35)	*p*
6^th^ h	3.44 ± 6.98	6.14 ± 12.31	0.126
12^th^ h	5.51 ± 10.71	8.86 ± 16.98	0.184
24^th^ h	1.36 ± 4.76	6.51 ± 14.60	0.003
2^nd^ day	0.55 ± 2.29	4.57 ± 11.27	0.005
3^th^ day	0.91 ± 4.42	4.71 ± 14.50	0.138
4^th^ day	0.36 ± 1.89	3.14 ± 11.05	0.135
5^th^ day	0.36 ± 1.9	2.29 ± 10.60	0.610
6^th^ day	0.18 ± 1.35	0.57 ± 3.38	0.734
7^th^ day	0.00 ± 0.00	0.29 ± 1.69	0.210

## Discussion

One of the distressing problems in endodontics is postoperative pain. This pain is caused by acute inflammation due to chemical, mechanical or microbial injury in the periapical tissues (Watkins, Logan & Kirchner, 2002; El Mubarak, Abu-bakr & Ibrahim, 2010; Sipavičiūtė & Manelienė, 2014; Wong et al., 2015) and may be associated with multiple factors namely patient gender, age, systemic comorbidities, preoperative pain, type of tooth and intracanal medicament and the presence of a periapical lesion (Ng et al., 2004).

In the present study, health-compromised patients were excluded to eliminate the effects of any systemic health-related factors on postoperative pain (Gondim et al., 2010; Tennert, Jungbäck & Wrbas, 2013; Nino-Barrera et al., 2018). Patients who used any analgesic, anti-inflammatory or antibiotic medication during the week preceding treatment were excluded to avoid potential bias caused by these drugs, as previously suggested by Comparin et al. (2017). Single-rooted teeth with a single root canal were selected to minimize the iatrogenic factors and differences caused by variations in root canal anatomy, similar to previous studies (Kandemir Demirci & Çalişkan, 2016; Gondim et al., 2010). In order to prevent bias, only vital pulp teeth were included, and all root canal treatments were completed in a single visit (Alonso-Ezpeleta et al., 2012; Gondim et al., 2010). When the treatments could not be completed in a single visit, patients were excluded from the study. Root canal treatments were postponed until the following clinic appointment in three patients. Reasons were patient related factors: such as feeling nausea or anxiety during the first appointment.

The existence of preoperative pain is a significant determinant of postoperative endodontic pain (El Mubarak, Abu-bakr & Ibrahim, 2010; Sadaf & Ahmad, 2014). Chronic preoperative pain can impact the balance of the central nervous system, thus even low-level stimuli can sensitize the nociceptors and trigger false pain signals (Seltzer & Naidorf, 2004). Therefore, patients with preoperative pain were not included in this study. It should be noted that pain is a subjective perception and, therefore, difficult to quantify. (Ng et al., 2004; Watkins, Logan & Kirchner, 2002; Sipavičiūtė & Manelienė, 2014) A variety of scales have been developed to assess postoperative pain. Although there are concerns regarding the VAS, it is a reliable and frequently used pain measurement method (Al-Negrish & Habahbeh, 2006; Jensen, Chen & Brugger, 2003). In this study, postoperative pain was assessed using a 100-mm VAS scale consisting of a 100 mm line, as previously suggested (Yaylali, Kurnaz & Tunca, 2018), to be conceptually easy and straightforward to administer.

Although age and general health of the patient have not been associated with root canal treatment success, they can affect the periapical tissue-healing process (Johnson et al., 2016). Jabeen & Khurshiduzzaman (2014) stated that older patients had more postoperative pain than younger patients. In contrast, Watkins, Logan & Kirchner (2002) found that younger patients anticipated more severe pain therefore suffered from more pain than older patients. With aging, the apical constriction (minor foramen) in root canals decreases the possibility of debris extrusion (Sipavičiūtė & Manelienė, 2014). Consequently, the frequency of flare-ups and postoperative pain is reduced. In addition, the reduced blood flow in the alveolar bone with age alleviates the inflammatory response of tissues (Sipavičiūtė & Manelienė, 2014). In line with this finding, the present study found an inverse relationship between age and postoperative pain. This relationship was significant at postoperative 6 h, 12 h and days 2 and 3. The participants of the study were adults of both genders. Like previous studies (Jabeen & Khurshiduzzaman, 2014; Al-Negrish & Habahbeh, 2006), no significant difference was found between gender and postoperative pain levels.

Herein, we evaluate the effects of different root canal obturation techniques following a single-visit root canal treatment on the manifestation of postoperative pain in patients with asymptomatic irreversible pulpitis or vital pulps. The null hypothesis of this study was that no difference in postoperative pain exists among the evaluated obturation techniques following a single-visit root canal treatment. Based on the results of this study, this hypothesis could be rejected. There were significant differences in the VAS scores among the groups at postoperative 6 h, 24 h and 2nd, 3rd and 4th days. Patients in the GuttaFlow2 group reported significantly lower VAS scores than those in CLC group during the 6 h post-treatment. Regarding other time points, however this difference was not significant. Higher pain level results in the CLC group at 6 h, would be an issue of the force applied by the spreader in the CLC technique, as reported by Wong et al. (2015)

Besides, patients in the GuttaFlow2 group had significantly lower mean postoperative pain levels after at postoperative 24 h and days 2, 3 and 4 (*i.e*., lower VAS scores) than the patients in the GuttaCore group. On the 3rd postoperative day, patients in the GuttaFlow2 and CLC groups reported significantly lower VAS scores compared with patients in the GuttaCore group. No difference was observed among the groups at postoperative 12 h and days 5, 6 and 7. In the first 12 h of the postoperative period, CLC resulted higher postoperative pain whereas in the later time periods GuttaCore demonstrated higher VAS scores. Alonso-Ezpeleta et al. (2012) likewise noted a relationship between the obturation technique and postoperative pain following root canal treatment. Specifically, Thermafil obturators, which belong to the same category as GuttaCore obturators, caused more postoperative pain at all follow-up intervals than the CLC and backfill techniques (Alonso-Ezpeleta et al., 2012). The higher pain levels were explained by the higher extrusion potential of the gutta-percha and the sealer in this technique.

The effect of overfilling of the root canal obturation material on postoperative pain, has baffled experts for years. Whereas some researchers have inferred that apical extrusion of filling material is responsible for postoperative pain (Sadaf & Ahmad, 2014; Fonseca et al., 2019), others still assert that it is caused by the extrusion of obturation material (Alonso-Ezpeleta et al., 2012; Kandemir Demirci & Çalişkan, 2016; Gondim et al., 2010; Yu et al., 2021). In the present study, extrusion of the filling material occurred in 4 (13.3%) cases in the CLC group, 9 (30.0%) cases in the GuttaFlow2 group and 22 (73.3%) cases in the GuttaCore group, respectively. In the GuttaCore group, the rate of filling material extrusion was significantly higher relative to the CLC group. The ratio of filling material extrusion in the CLC group was similar to previous studies (Kandemir Demirci & Çalişkan, 2016; Tennert, Jungbäck & Wrbas, 2013). The results of this study corroborate previous research in which the ratio of filling material extrusion was relatively higher after the Thermafil obturation technique (Kandemir Demirci & Çalişkan, 2016; Tennert, Jungbäck & Wrbas, 2013). Although the obturation material extruded in nine cases in the GuttaFlow2 group, it was noted that patients in this group did not have pain after the 2^nd^ postoperative day, which is a much shorter period than when the CLC and GuttaCore techniques were applied. We believe that the absence of postoperative pain in this group could be because of the low cytotoxicity, high biocompatibility, and compaction-free properties of GuttaFlow2 (Accardo, Himel & Lallier, 2014; Mandal et al., 2014).

Besides, unintentional extrusion of sealer through the apical constriction may cause irritation, inflammation and a possible delay in wound-healing, along with matrix protein degeneration (Silva et al., 2015; Huang, Yang & Chang, 2008). It is known that matrix metalloproteinase-2 (MMP-2) expression may induce extracellular matrix proteolysis and lead to the inflammatory process (Huang, Yang & Chang, 2008). Sealers may activate MMPs, which play an important role in the pathogenesis of root canal sealer-induced periapical inflammation (Accardo, Himel & Lallier, 2014). A study comparing the cytotoxicity and gelatinolytic activity of GuttaFlow2 and AH Plus concluded that GuttaFlow2 was non-toxic and did not affect MMP-2 levels (Silva et al., 2015). As a result, GuttaFlow2 was proposed as a more biocompatible root canal sealer than its resin-based compartment, AH Plus. Likewise, these attributes of the GuttaFlow2 obturation technique are probably responsible for causing the shorter period of postoperative pain as observed in our study. Our observation of a significant relationship between pain on the 1^st^ and 2^nd^ post obturation days and the presence of filling material extrusion, concurs with previous reports (Alonso-Ezpeleta et al., 2012; Kandemir Demirci & Çalişkan, 2016; Nino-Barrera et al., 2018). Extruded filling materials may cause pain by changing the pressure in the periapical region and trigger the inflammatory response (Sathorn, Parashos & Messer, 2008). Therefore, relatively higher postoperative pain levels in the GuttaCore group may be ascribed to the immunological reaction triggered by extruded root canal filling materials and the pressure changes in the periapical region (Accardo, Himel & Lallier, 2014; Mandal et al., 2014).

Similar to the studies of Nekoofar et al. (2015) and Yaylali, Kurnaz & Tunca (2018), an analgesic (ibuprofen) was prescribed. The patients were asked to use it only when they had severe pain and to record the time of drug intake as previously suggested by Keskin et al. (2017). In the present study, no significant difference was observed among the groups in terms of analgesic intake frequency, this is similar to a previous research (Aslan & Dönmez Özkan, 2021). In contrast, Atav Ates et al. (2019) declared that iRoot SP sealer was associated with less analgesic intake compared to AH Plus sealer in their randomized controlled clinical trial. The differences between the results of these studies in terms of analgesic intake might be related with the different chemical properties of the tested sealer.

Our study has some limitations. Despite all the precautions taken, it is not always possible to control all potential sources of pain, and there are individual differences that may lead to bias. Moreover, operator skills are one of the other potential bias factors. Although a single operator was performed all the treatments in this study, being familiar or not with the used obturation technique may have affected operator skills. As previously reported, the possibility of overfilling by thermoplastic obturation techniques is four-fold higher in anterior teeth than posterior teeth (Nino-Barrera et al., 2018). In addition, because only single-rooted teeth with one canal were investigated in this study, caution is needed before generalizing the results to multi-rooted teeth. Although apical enlargement size is critical for postoperative pain, it was not possible to obtain standardized apical size under clinical conditions. Therefore, it should be considered one of the significant limitations of the study.

## Conclusions

Within the limitations of this study, GuttaCore demonstrated higher pain scores at all time periods except during the first 12 h. On the other hand, GuttaFlow2 provided lower or similar postoperative pain compared to CLC. To the best of our knowledge, this is the first study to investigate the effects of the cold free-flow compaction technique (GuttaFlow2) on postoperative pain. Further investigations on both single- and multi-rooted teeth are required to confirm its global effect on postoperative pain following root canal treatments.

## Supplemental Information

10.7717/peerj.13756/supp-1 Supplemental Information 1 Raw Data.

10.7717/peerj.13756/supp-2 Supplemental Information 2 Consort Checklist.

## Additional Information and Declarations

### Data Availability

The following information was supplied regarding data availability: The raw measurements are available as a Supplemental File.

### Clinical Trial Registration

The following information was supplied regarding Clinical Trial registration: NCT/04228913.

## References

[ref-1] Accardo C, Himel VT, Lallier TE (2014). A novel GuttaFlow sealer supports cell survival and attachment. Journal of Endodontics.

[ref-2] Al-Negrish AR, Habahbeh R (2006). Flare up rate related to root canal treatment of asymptomatic pulpally necrotic central incisor teeth in patients attending a military hospital. Journal of Dentistry.

[ref-3] Alonso-Ezpeleta LO, Gasco-Garcia C, Castellanos-Cosano L, Martin-Gonzalez J, Lopez-Frias FJ, Segura-Egea JJ (2012). Postoperative pain after one-visit root-canal treatment on teeth with vital pulps: comparison of three different obturation techniques. Medicina Oral, Patologia Oral, Cirugia Bucal.

[ref-4] Aslan T, Dönmez Özkan H (2021). The effect of two calcium silicate-based and one epoxy resin-based root canal sealer on postoperative pain: a randomized controlled trial. International Endodontic Journal.

[ref-5] Atav Ates A, Dumani A, Yoldas O, Unal I (2019). Post-obturation pain following the use of carrier-based system with AH Plus or iRoot SP sealers: a randomized controlled clinical trial. Clinical Oral Investigations.

[ref-6] Collado-González M, Tomás-Catalá CJ, Oñate-Sánchez RE, Moraleda JM, Rodríguez-Lozano FJ (2017). Cytotoxicity of GuttaFlow Bioseal, GuttaFlow2, MTA Fillapex, and AH Plus on human periodontal ligament stem cells. Journal of Endodontics.

[ref-7] Comparin D, Moreira EJL, Souza EM, De-Deus G, Arias A, Silva ENL (2017). Postoperative pain after endodontic retreatment using rotary or reciprocating instruments: a randomized clinical trial. Journal of Endodontics.

[ref-8] El Mubarak AH, Abu-bakr NH, Ibrahim YE (2010). Postoperative pain in multiple-visit and single-visit root canal treatment. Journal of Endodontics.

[ref-9] Fonseca B, Coelho MS, Bueno CS, Fontana CE, Martin ASD, Rocha DGP (2019). Assessment of extrusion and postoperative pain of a bioceramic and resin-based root canal sealer. European Journal of Dentistry.

[ref-10] Gondim E, Setzer FC, dos Carmo CB, Kim S (2010). Postoperative pain after the application of two different irrigation devices in a prospective randomized clinical trial. Journal of Endodontics.

[ref-11] Gutmann JL, Saunders WP, Saunders EM, Nguyen L (1993). An assessment of the plastic Thermafil obturation technique. Part 1. Radiographic evaluation of adaptation and placement. International Endodontic Journal.

[ref-12] Huang FM, Yang SF, Chang YC (2008). Up-regulation of gelatinases and tissue type plasminogen activator by root canal sealers in human osteoblastic cells. Journal of Endodontics.

[ref-13] Jabeen S, Khurshiduzzaman M (2014). Incidence of post obturation pain following single and multivisit root canal treatment in a teaching hospital of Bangladesh. Mymensingh Medical Journal.

[ref-14] Jensen MP, Chen C, Brugger AM (2003). Interpretation of visual analog scale ratings and change scores: a reanalysis of two clinical trials of postoperative pain. Journal of Pain.

[ref-15] Johnson WT, Kulild JC, Tay F, Hargreaves KM, Berman LH, Hargreaves KM, Berman LH (2016). Obturation of the cleaned and shaped root canal system. Cohen’s Pathways of the Pulp.

[ref-16] Kandemir Demirci G, Çalişkan MK (2016). A prospective randomized comparative study of cold lateral condensation versus core/gutta-percha in teeth with periapical lesions. Journal of Endodontics.

[ref-17] Keskin C, Özdemir Ö, Uzun İ, Güler B (2017). Effect of intracanal cryotherapty on pain after single-visit root canal treatment. Australian Endodontic Journal.

[ref-18] Lea C, Apicella M, Mines P, Yancich P, Parker M (2005). Comparison of the obturation density of cold lateral compaction versus warm vertical compaction using the continuous wave of condensation technique. Journal of Endodontics.

[ref-19] Mandal P, Zhao J, Sah SK, Huang Y, Liu J (2014). In vitro cytotoxicity of guttaflow 2 on human gingival fibroblasts. Journal of Endodontics.

[ref-20] Mohan Kumar NS, Prabu PS, Prabu N, Rathinasamy S (2012). Sealing ability of lateral condensation, thermoplasticized gutta-percha and flowable gutta-percha obturation techniques: a comparative in vitro study. Journal of Pharmacy and Bioallied Sciences.

[ref-21] Nekoofar MH, Sheykhrezae MS, Meraji N, Jamee A, Shirvani A, Jamee J, Dummer PMH (2015). Comparison of the effect of root canal preparation by using WaveOne and ProTaper on postoperative pain: a randomized clinical trial. Journal of Endodontics.

[ref-22] Nevares G, de Albuquerque DS, Bueno CE, Cunha RS (2015). Is GuttaCore more easily removed from the root canal than Thermafil? An ex-vivo study. Journal of Canadian Dental Association.

[ref-23] Ng Y-L, Glennon JP, Setchell DJ, Gulabivala K (2004). Prevalence of and factors affecting post-obturation pain in patients undergoing root canal treatment. International Endodontic Journal.

[ref-24] Nino-Barrera JL, Gamboa-Martinez LF, Laserna-Zuluaga H, Unapanta J, Hernández-Mejia D, Olaya C, Alzate-Mendoza D (2018). Factors associated to apical overfilling after a thermoplastic obturation technique - Calamus® or Guttacore®: a randomized clinical experiment. Acta Odontológica Latinoamericana.

[ref-25] Orstavik D (2010). Materials used for root canal obturation: technical, biological and clinical testing. Endodontic Topics.

[ref-26] Punjabi M, Dewan RG, Kochhar R (2017). Comparative evaluation of fracture resistance of root canals obturated with four different obturating systems. Journal of Conservative Dentistry.

[ref-27] Sadaf D, Ahmad MZ (2014). Factors associated with postoperative pain in endodontic therapy. International Journal of Biomedical Science.

[ref-28] Sathorn C, Parashos P, Messer H (2008). The prevalence of postoperative pain and flare-up in single-and multiple-visit endodontic treatment: a systematic review. International Endodontic Journal.

[ref-29] Saygili G, Saygili S, Tuglu I, Capar ID (2017). In vitro cytotoxicity of GuttaFlow Bioseal, GuttaFlow 2, AH-Plus and MTA Fillapex. Iranian Endodontic Journal.

[ref-30] Seltzer S, Naidorf IJ (2004). Flare-ups in endodontics: I. Etiological factors. Journal of Endodontics.

[ref-31] Silva EJNL, Neves AA, De-Deus G, Accorsi-Mendonça T, Moraes AP, Valentim RM, Moreira EJ (2015). Cytotoxicity and geletinolytic activity of a new silicon-based endodontic sealer. The Journal of Applied Biomaterials & Functional Materials.

[ref-32] Sipavičiūtė E, Manelienė R (2014). Pain and flare-up after endodontic treatment procedures. Stomatologija, Baltic Dental and Maxillofacial Journal.

[ref-33] Sjögren U, Hägglund B, Sundqvist G, Wing K (1990). Factors affecting the long-term results of endodontic treatment. Journal of Endodontics.

[ref-34] Tennert C, Jungbäck IL, Wrbas KT (2013). Comparison between two thermoplastic root canal obturation techniques regarding extrusion of root canal filling--a retrospective in vivo study. Clinical Oral Investigation.

[ref-35] Wang C, Xu P, Ren L, Dong G, Ye L (2010). Comparison of post-obturation pain experience following one-visit and two-visit root canal treatment on teeth with vital pulps: a randomized controlled trial. International Endodontic Journal.

[ref-36] Watkins CA, Logan HL, Kirchner HL (2002). Anticipated and experienced pain associated with endodontic therapy. Journal of American Dental Association.

[ref-37] Wong AW-Y, Zhang S, Li SK-Y, Zhu X, Zhang C, Chu C-H (2015). Incidence of post-obturation pain after single-visit vs. multiple-visit non-surgical endodontic treatments. BMC Oral Health.

[ref-38] Yaylali IE, Kurnaz S, Tunca YM (2018). Maintaining apical patency does not increase postoperative pain in molars with necrotic pulp and apical periodontitis: a randomized controlled trial. Journal of Endodontics.

[ref-39] Yu Y-H, Kushnir L, Kohli M, Karabucak B (2021). Comparing the incidence of postoperative pain after root canal filling with warm vertical obturation with resin-based sealer and sealer-based obturation with calcium silicate-based sealer: a prospective clinical trial. Clinical Oral Investigations.

[ref-40] Zielinski TM, Baumgartner JC, Marshall JG (2008). An evaluation of Guttaflow and gutta-percha in the filling of lateral grooves and depressions. Journal of Endodontics.

